# Implementation of computerised physician order entry (CPOE) and picture archiving and communication systems (PACS) in the NHS: quantitative before and after study

**DOI:** 10.1136/bmj.a939

**Published:** 2008-08-14

**Authors:** Simon Collin, Barnaby C Reeves, Jane Hendy, Naomi Fulop, Andrew Hutchings, Eugenia Priedane

**Affiliations:** 1 Department of Social Medicine, University of Bristol; 2 Department of Clinical Sciences at South Bristol, University of Bristol, Bristol; 3 Health and Care Infrastructure Research and Innovation Centre, Imperial College, London; 4 Health and Health Policy NIHR King’s Patient Safety and Service Quality Research Centre, King’s College, London; 5 Department of Public Health and Policy, London School of Hygiene and Tropical Medicine

## Abstract

**Objective** To assess the impact of components of the national programme for information technology (NPfIT) on measures of clinical and operational efficiency.

**Design** Quasi-experimental controlled before and after study using routinely collected patient level data.

**Setting** Four NHS acute hospital trusts in England.

**Data sources** Inpatient admissions and outpatient appointments, 2000-5.

**Interventions** A system for ordering pathology tests and browsing results (computerised physician order entry, CPOE) and a system for requesting radiological examinations and displaying images (picture archiving and communications system, PACS).

**Main outcome measures** Requests per inpatient, outpatient, or day case patient for full blood count, urine culture and urea and electrolytes tests, and plain x ray film, computed tomography, and ultrasonography examinations.

**Results** CPOE was associated with a reduction in the proportion of outpatient appointments at which full blood count (odds ratio 0.25, 95% confidence interval 0.16 to 0.40), urea and electrolytes (0.55, 0.39 to 0.77), and urine culture (0.30, 0.17 to 0.51) tests were ordered, and at which full blood count tests were repeated (0.73, 0.53 to 0.99). Conversely, the same system was associated with an almost fourfold increase in the use of urea and electrolytes tests among day case patients (3.63, 1.66 to 7.94). PACS was associated with a reduction in repeat plain x ray films at outpatient appointments (0.62, 0.44 to 0.88) and a reduction in inpatient computed tomography (0.83, 0.70 to 0.98). Conversely, it was associated with increases in computed tomography requested at outpatient appointments (1.89, 1.26 to 2.84) and computed tomography repeated within 48 hours during an inpatient stay (2.18, 1.52 to 3.14).

**Conclusions** CPOE and PACS were associated with both increases and reductions in tests and examinations. The magnitude of the changes is potentially important with respect to the efficiency of provision of health care. Better information about the impact of modern IT is required to enable healthcare organisations to manage implementation optimally.

## Introduction

The rate at which information technology (IT) systems are being ordered and deployed by healthcare providers around the world has far outpaced the growth of the evidence base of clinical and operational benefits associated with such systems. This is particularly so for the installation of large scale commercial systems in hospitals that provide acute care and outpatient services across a wide range of clinical specialties. Two recent systematic reviews that assessed the impact of healthcare IT concluded that, although the theoretical benefits remained clear, further research into actual gains was urgently needed.^1^ ^2^ In the United Kingdom, an estimated £20bn over 10 years is being invested in the National Health Service (NHS) national programme for information technology (NPfIT).^3^ This programme is expected to yield improvements in the quality of clinical care and operational efficiency.^4^

We previously reported the findings of a qualitative study that assessed challenges and progress in implementing NPfIT in four NHS acute hospital trusts in England by means of interviews with managers and clinicians.^5^ ^6^ Here we report the findings of a quantitative study conducted in the same trusts in parallel with the qualitative study. We assessed the impact on measures of clinical and operational efficiency of IT systems, including the Choose and Book electronic referral system, implemented under the auspices of NPfIT. Because of delays in the national programme, however, we based our final assessment on the implementation of a system for ordering pathology tests and browsing results (referred to here as computerised physician order entry or CPOE) and a system for requesting radiological examinations and storing and displaying images (referred to as a picture archiving and communications system or PACS). Although such systems are key components of NPfIT, both systems in our study were implemented independently of NPfIT.

## Methods

### Study design

We selected four trusts representing a range of characteristics of NHS hospital trusts (size, financial situation, and state of information technology development). We used a quasi-experimental “controlled before and after cohort” design,^7^ with each trust as a unit of the experiment, to quantify the effects of IT systems implemented in 2000-5. We divided this period into before, during, and after implementation, depending on when the system was implemented. We retrospectively observed implementation of CPOE, PACS, and other systems in any of the participating trusts. One trust (trust 1) implemented CPOE and one (trust 4) implemented PACS (table 1). By coincidence, the implementation periods were the same (2001-2). We quantified associations of implementation with outcome by comparing outcomes during the before and after periods in the trust that implemented the system with outcomes during the same periods in the three trusts that did not implement the system. For the CPOE analysis, we considered three types of pathology test: full blood count, urea and electrolytes, and urine culture. For the PACS analysis, we considered three types of radiological examination: plain film, computed tomography, and ultrasonography.

**Table 1 tbl1:** Characteristics of participating trusts

	Trust 1	Trust 2	Trust 3	Trust 4
No of beds	954 (2 sites)*	821 (2 sites)†	1110 (1 site)	470 (1 site)
Forecast cumulative deficit (1997-2007) (% of 2006-7 turnover)	£38m (14.5%)	£67m (26.0%)	£14m (3.7%)	£1.5m (1.1%)
CPOE	Implemented at one site 2001-2‡	None	None	None
PACS	None	None	None	Implemented 2001-2§
Average annual inpatient admissions	78 673	75 918	102 217	34 399
Average annual outpatient appointments	394 979	396 442	411 763	198 969
Average annual pathology tests (full blood count, urea and electrolytes, and urine culture) for inpatients, outpatients and A&E¶	187 541	242 030	352 675	310 242
Average annual radiological examinations (plain film, CT, and ultrasonography) for inpatients, outpatients and A&E	77 934	192 856	172 757	72 806

CPOE=computerised physician order entry; PACS=picture archiving and communications system; A&E=accident and emergency department; CT=computed tomography.

*No inpatient, outpatient, radiology, or pathology data were available for one of two sites, hence analyses based on data from single site within this trust.

†Trust 2 analysed as single entity because both sites were managed by same patient administration system.

‡Except in maternity.

§First in A&E and trauma and orthopaedics, then in all other specialties—excludes ultrasonography and mammography.

¶Urea and electrolytes test data unavailable for trust 2.

### Outcomes

Our outcomes were proxy measures of clinical and operational efficiency derived from a larger set of indicators that had been defined a priori, based partly on consideration of the NHS efficiency map.^4^ We classified outcomes as primary or secondary depending on whether a direct causal pathway between implementation of an IT system and the outcome was plausible or not (see table A on bmj.com). Primary outcomes for inpatients were investigations (pathology test orders or radiological examination requests) per inpatient, investigations per day case, and investigation within 48 hours of previous investigation of the same type; primary outcomes for outpatients were investigation at outpatient appointment and same investigation at next outpatient appointment. Secondary outcomes for inpatients were length of stay, day cases as a proportion of admitted patients, ratio of actual to intended day cases, emergency readmission, death, and time to death; secondary outcomes for outpatients were attended/did not attend and outcome of appointment (discharged *v* follow-up). We refer to changes in outcomes as “efficiency gains” where we consider the change to reflect an improvement in clinical or operational efficiency—for example, a reduction in the number of pathology test orders—and as “detrimental” if the opposite.

### Data sources

Inpatient and outpatient datasets for 2000-5 were subsets of NHS commissioning datasets (CDS) obtained from information management and technology departments in each trust. Pathology and radiology data, documenting all tests and examinations carried out during the same period, were obtained from relevant departments. All datasets contained a local patient identifier, which we used to join the inpatient and outpatient datasets with the pathology and radiology datasets and so derive the primary outcomes. Secondary outcomes were derived directly from the inpatient and outpatient data.

### Data analysis

We analysed the records of individual patients for a range of specialties for which care was provided by all four trusts. For inpatients, these included general surgery, general medicine, urology, trauma and orthopaedics, accident and emergency, paediatrics, and obstetrics and gynaecology. Common specialties for outpatients included all of the inpatient specialties plus otorhinolaryngology, ophthalmology, endocrinology, haematology, cardiology, dermatology, nephrology, oncology, neurology, rheumatology, and geriatric medicine.

We estimated effects by multiple regression modelling, calculating robust standard errors to take into account clustering of individual records by the common specialties (seven inpatient, 18 outpatient) within the four trusts, resulting in 28 clusters for inpatient data and 72 clusters for outpatient data. Effects on binary outcomes were assessed with logistic regression, and effects on continuous outcomes by ordinary least squares linear regression, logarithmically transformed to obtain a near normal distribution. We analysed continuous outcomes with a high proportion of zero values using logistic regression to model the probability of a zero response and linear regression to model the non-zero continuous response.^8^ We assessed effects on length of stay and time to death by Cox regression, after checking the proportional hazards assumption. In each regression model, the effect of implementation of CPOE or PACS was estimated by including a term for interaction between the intervention (trust) and the time period, specifically by the regression coefficient of the interaction parameter corresponding to the period after the intervention. We report exponents of these regression coefficients—that is, odds or hazards ratios and relative changes in continuous outcomes. All analyses were performed with Stata v9.2 (StataCorp, College Station, TX).

## Results

### Participating trusts and systems implementation

Table 1 presents background information about each trust in the study and shows which of the trusts implemented CPOE or PACS.[Table tbl1]

Trust 1 was the only trust to implement a CPOE system. This system provided test ordering (with automated form filling, order sets, warnings of possible test duplication, and user defined rules) and access to previous test results. In the three trusts without CPOE, some form of computer based access to results of pathology tests tended to be available but was not widely or consistently used by clinicians. Trust 2 had tried but failed to implement CPOE.

Trust 4 was the only trust to implement PACS. This system provided web based access to requested and archived images and was implemented together with a new (but separate) system for requesting examinations. Trusts 2 and 3 had limited PACS functionality, implemented before the period covered by our study: trust 2 in a daycare unit comprising two procedures (magnetic resonance imaging and fluoroscopy) not included in our study and trust 3 in its children’s hospital (x ray pictures only, mainly within intensive care). 

Trust 2 was unable to provide pathology data for the period before October 2002, and no data on urea and electrolyte tests were available for this trust. Data for the first three months of year 2000 were missing for inpatients and outpatients in trust 2, pathology in trust 3, and pathology and radiology in trust 4. No data were available for one of the two sites in trust 1.

### CPOE primary outcomes

Table 2 summarises the results of the comparisons for implementation of CPOE.[Table tbl2] This table shows the changes (after minus before) in the outcomes in intervention and control trusts and the effects of implementing the CPOE system as the relative increase or decrease in each outcome (estimated by interactions of implementation and period in the regression models), adjusted for underlying trends in all trusts. See table B on bmj.com for the data on which these analyses are based.

**Table 2 tbl2:** Implementation of CPOE in trust 1 compared with trusts 2, 3, and 4. Figures are odds ratios, or regression coefficients where specified (95% confidence intervals) for interaction between intervention (in trust 1) and period after intervention (2003-5) and mean change for intervention trust *v* control trusts

Primary outcomes*	Full blood count	Urea and electrolytes†	Urine culture
**Inpatient**			
Tests per inpatient: non-zero *v* zero response	0.74 (0.48 to 1.16)	0.66 (0.43 to 1.02)	1.14 (0.80 to 1.63)
Change	1.9%* v* 1.1%	7.8% *v* 5.3%	−4.3% *v* 3.7%
Tests per inpatient day: continuous non-zero response	1.00‡ (0.90 to 1.10)	1.03‡ (0.89 to 1.18)	0.93‡ (0.82 to 1.06)
Change	0.05 *v* 0.03	0.08 *v* 0.05	−0.01 *v* 0.05
Tests per day case: non-zero *v* zero response	1.76 (0.78 to 3.99)	3.63 (1.66 to 7.94)	1.29 (0.54 to 3.13)
Change	6.5% *v* 2.2%	8.0% *v* 5.9%	1.9% *v* 1.2%
Test within 48 hours of previous test of same type (inpatients)	0.93 (0.79 to 1.10)	1.07 (0.89 to 1.29)	0.89 (0.70 to 1.12)
Change	1.6% *v* 3.8%	−0.2% *v* 0.5%	−1.4% *v* −0.1%
**Outpatient**			
Test(s) at outpatient appointment	0.25 (0.16 to 0.40)	0.55 (0.39 to 0.77)	0.30 (0.17 to 0.51)
Change	−1.9% *v* 4.6%	−0.6% *v* 3.6%	−0.5% *v* 1.5%
Test of same type at next outpatient appointment	0.73 (0.53 to 0.99)	0.84 (0.64 to 1.11)	0.73 (0.52 to 1.02)
Change	0.6% *v* 4.3%	4.3% *v* 6.0%	0.4% *v* 2.3%

*See table B on bmj.com for full data for each trust.

†No data contributed by trust 2.

‡Exponent of regression coefficient.

Evidence for possible efficiency gains due to implementation of CPOE was most apparent in the reduction in outpatient tests. This effect was seen for full blood count, urea and electrolytes, and urine culture tests, and was due to decreases in the numbers of each of these tests ordered at outpatient appointments in the intervention trust, compared with increases in the two control trusts for which data were available. There was also an effect of CPOE in reducing “repeat” full blood count tests at outpatient appointments, which was due to larger increases in this measure in the control trusts compared with the intervention trust.

Conversely, CPOE was associated with an almost fourfold increase in the use of urea and electrolytes tests among day case patients. This effect was due to a large increase in this indicator in trust 1 (from 2.2% to 10.2%) compared with the two control trusts, one of which also saw a large increase (from 9.9% to 18.7%).

### PACS primary outcomes

Table 3 summarises the effects of implementing PACS[Table tbl3] (see table C on bmj.com for the data on which these analyses were based). Evidence for possible efficiency gains due to implementation of PACS was apparent in the reduction in repeat plain x ray film exams at outpatient appointments and in the reduction in inpatient computed tomography. The first effect was due to a slight decrease in the intervention trust compared with increases in the three control trusts. The second effect was due to a relatively small increase in inpatient computed tomography in the intervention trust 4 (from 8.1% to 10.2%) compared with larger increases in the control trusts.

**Table 3 tbl3:** Implementation of PACS in trust 4 compared with trusts 1, 2, and 3. Figures are odds ratios, or regression coefficients where specified (95% confidence intervals) for interaction between intervention (in trust 4) and period after intervention (2003-5) and mean change for intervention trust *v* control trusts

Primary outcomes*	Plain x ray film	Computed tomography	Ultrasonography†
**Inpatient**	****	****	****
Exams per inpatient: non-zero *v* zero response	0.90 (0.71 to 1.14)	0.83 (0.70 to 0.98)	0.89 (0.69 to 1.14)
Change	1.0% *v* 4.1%	2.1% *v* 3.0%	−1.3% *v* 0.5%
Exams per inpatient day: continuous non-zero response	0.97‡ (0.90 to 1.05)	1.02‡ (0.91 to 1.14)	0.96‡ (0.85 to 1.09)
Change	0.02 *v* 0.02	0.02 *v* 0.05	−0.01 *v* 0.00
Exams per day case: non-zero *v* zero response	1.01 (0.55 to 1.86)	0.73 (0.31 to 1.73)	1.55 (0.83 to 2.89)
Change	7.0% *v* 5.2%	0.7% *v* 0.7%	0.0% *v* −0.2%
Exam within 48 hours of previous exam of same type (inpatients)	1.02 (0.91 to 1.14)	2.18 (1.52 to 3.14)	1.08 (0.81 to 1.44)
Change	−3.2% *v* −4.3%	1.2% *v* −0.1%	0.2% *v* 0.2%
**Outpatient**	****	****	****
Exam(s) at outpatient appointment	0.90 (0.76 to 1.07)	1.89 (1.26 to 2.84)	1.48 (0.60 to 3.66)
Change	1.0% *v* 0.0%	0.2% *v* 0.1%	1.9% *v* −0.1%
Exam of same type at next outpatient appointment	0.62 (0.44 to 0.88)	NA	0.58 (0.19 to 1.82)
Change	−1.2% *v* 4.6%	NA	−10.4% *v* −2.2%

NA=not analysed because of insufficient numbers.

*See table C on bmj.com for full data for each trust.

†Ultrasonography not included in PACS in intervention trust (trust 4).

‡Exponent of regression coefficient.

Conversely, implementation of PACS was associated with increases in computed tomography requested at outpatient appointments and computed tomography repeated within 48 hours during an inpatient stay. The first effect was due to a big increase in outpatient computed tomography in the intervention trust (from 0.02% to 0.21%) compared with no change in trusts 2 and 3, although there was a similarly big increase in trust 1 (from 0.03% to 0.25%). The second effect derived from a doubling of repeat inpatient computed tomography in trust 4 (from 1.2% to 2.4%) compared with small reductions in trusts 2 and 3 and a slight increase in trust 1. Ultrasonography was not a component of the PACS in trust 4, and there was no evidence of changes in outcomes.

### Secondary outcomes

Table 4 presents the results of our analyses of the impact of CPOE and PACS on secondary outcomes[Table tbl4] (see table D on bmj.com for the data on which these analyses were based). The comparisons showed evidence of detrimental effects of CPOE and PACS in reducing the proportion of outpatients discharged, a detrimental effect of CPOE in reducing outpatient attendance, and a beneficial impact of CPOE in reducing inpatient deaths.

**Table 4 tbl4:** Implementation of CPOE (trust 1) and PACS (trust 4); odds ratios, or hazard ratios where specified (95% confidence intervals) for interaction between intervention (in trust 1 or trust 4) and period after intervention (2003-5) and mean change for intervention trust *v* control trusts

Secondary outcomes*	CPOE	PACS
**Inpatient**		
Length of stay (days) (excluding day cases)	1.02† (0.96 to 1.08)	0.95† (0.89 to 1.02)
Change	−0.29 *v* −0.40	0.30 *v* −0.40
Likelihood of being a day case	0.97 (0.77 to 1.22)	0.92 (0.74 to 1.15)
Change	4.3% *v* 6.2%	2.7% *v* 6.2%
Intended day cases admitted overnight	NA	0.85 (0.53 to 1.39)
Change	NA	−3.0% *v* −3.4%
Emergency re-admission (within 28 days)	1.05 (0.84 to 1.32)	0.95 (0.79 to 1.14)
Change	1.3% *v* 1.1%	1.5% *v* 1.1%
Deaths	0.82 (0.71 to 0.95)	0.91 (0.75 to 1.09)
Change	−0.3% *v* 0.1%	−0.3% *v* 0.1%
Time to death (days)	0.98† (0.92 to 1.04)	1.05† (0.99 to 1.11)
Change	2.30 *v* 1.15	0.90 *v* 1.15
**Outpatient**		
Attendance (attended *v* did not attend)	0.87 (0.78 to 0.98)	0.94 (0.86 to 1.04)
Change	0.2% *v* 2.2%	1.5% *v* 2.2%
Outcome (discharged *v* follow-up)	0.73 (0.55 to 0.98)	0.58 (0.43 to 0.78)
Change	2.6% *v* 5.1%	-3.4% *v* 5.1%

NA=no data available.

*See table D on bmj.com for full data for each trust.

†Hazard ratio.

## Discussion

Two IT systems showed both benefit and detriment on various efficiency outcomes. The main strength of our study is its scale and its scope, which far surpass previous studies, both in the UK and internationally. We analysed data over a five year period from four English NHS trusts, each of which comprised at least one large hospital. We evaluated two different systems, CPOE and PACS, each comprising three different procedures (test type in CPOE, examination type in PACS) and analysed a range of primary and secondary outcomes related to inpatient and outpatient care. Our study was made possible by the uniformity of data reporting across NHS trusts. This allowed us to join patient administration data with data from pathology and radiology departments. We found evidence for an effect of CPOE on five out of 18 primary outcomes and on three out of seven secondary outcomes; and for PACS, on four of 17 primary outcomes and one of eight secondary outcomes. Of the five effects on primary outcomes attributable to CPOE, four were indicative of efficiency gains; for PACS, two out of four.

### Impact of CPOE on primary outcomes

The reduction in outpatient tests could plausibly be attributed to implementation of CPOE. The CPOE system enabled clinicians to access the patient’s pathology test history during an outpatient appointment, which could have reduced the number of tests ordered because of a missing previous test result. This argument is strengthened by the reduction in repeat full blood counts ordered at consecutive outpatient appointments. Reasons for the large increase in urea and electrolytes test ordering across all trusts, and for the greater relative increase in ordering of this test in the intervention trust, were not apparent.

In a recent systematic review, CPOE was associated with a reduced volume of pathology tests in seven out of 11 studies, with no change in three studies, and with an increase in one study.^2^ Only one of the studies (showing reduced volume) was performed in outpatient departments, and the intervention evaluated in this study was a module added to an existing CPOE system to display test charges.^9^ We found one additional study in a US primary care setting, which reported a reduction in ordering of six types of pathology tests (including full blood count, urea and electrolytes, and urine culture) if previous test results were displayed.^10^ This result was for all tests combined; the slight decrease in orders for full blood count and urea and electrolytes tests was not analysed separately and orders for urine culture test showed a slight increase.^10^

### Impact of PACS on primary outcomes

Implementation of PACS was associated with fewer computed tomograms being requested for inpatients but more scans requested at outpatient appointments. PACS was also associated with an increase in computed tomography repeated within 48 hours during inpatient stay. Possible explanations for the relatively large increases in outpatient computed tomography and repeat inpatient scans in the intervention trust, and the large increase in outpatient scans in trust 1, were not forthcoming from the trusts. New scanners were installed in the intervention trust in 2000 and 2006. A new scanner was installed in trust 1 in 2003, which replaced an existing machine. These results suggest that implementation of PACS in the intervention trust allowed an increasing demand for computed tomography to be met through outpatient appointments, rather than through inpatient admissions, possibly as a consequence of shorter turnaround times (from examination request to image availability).^11^ It is then plausible that those patients who still required hospital admission would be those patients who needed repeat scans. A large increase in outpatient computed tomography, however, was also seen in one of the control trusts, hence attribution of these effects to implementation of PACS is questionable. PACS was also associated with fewer repeat plain x ray film exams at consecutive outpatient appointments. As with repeat full blood counts at consecutive outpatient appointments, attribution of this effect to implementation of PACS is plausible if PACS enables the clinician to access the patient’s radiological examination history during the outpatient appointment. Fewer repeats could also be related to lower rejection rates.^12^

### Impact of CPOE and PACS on secondary outcomes

There seemed to be a consistent reduction in the proportion of patients discharged at outpatient appointments after CPOE and PACS were implemented, but we found no explanation for this apparently detrimental effect. Neither could we explain the reductions in deaths or outpatient appointment attendances associated with CPOE. Attribution of changes in secondary outcomes due to implementation of CPOE or PACS was problematic because the hypothesised chain of causality linking implementation of CPOE or PACS to secondary outcomes was more tenuous than for primary outcomes. Studies on length of stay found no impact of CPOE^2^ or PACS,^13^ ^14^ ^15^ except for one that found a reduction associated with implementation of PACS.^11^ Secondary outcomes were more likely to be influenced by major process changes within each trust. For example, deployment of CPOE within trust 1 coincided with construction of a new hospital under a government private finance initiative (PFI). Such trust-wide changes would influence our estimates if they occurred differentially in intervention and control trusts and if they coincided with implementation of CPOE and PACS. The absence of effects of CPOE and PACS on most of our secondary outcomes could be interpreted as evidence that the effects that we observed on our primary outcomes were not confounded by process changes throughout the trust.

### Study in context

CPOE in trust 1 and PACS in trust 4 were considered by managers and end users to have been successful implementations of these types of healthcare IT system,^16^ preceding by several years the rollout of similar systems under NPfIT. The NHS is leading the way in terms of the scale and homogeneity of its healthcare IT programme and, although running behind schedule and over budget, the programme continues to receive the support of managers and clinicians alike.^5^ ^6^ CPOE and PACS, when fully integrated with the other information technology systems that comprise NPfIT (national electronic health records, patient administration systems, electronic referral, etc), might contribute to more dramatic quantitative changes.

Our study has shown that it is possible to use routinely collected patient level data from disparate sources within large healthcare institutions as a basis for assessing the impact of technological changes on indicators of clinical activity and operational efficiency. In the context of future research, the transmission of local (trust level) patient identifiers in commissioning datasets to the NHS-wide clearing service and the ready availability of datasets from specialist departments within trusts suggest that our technique of joining data from commissioning datasets with these specialist datasets could form the basis for operational research on a nationwide scale. Use of NHS patient numbers to identify patients uniquely might improve the quality of such an exercise. The use of intervention and control groups comprising a much larger number of trusts would improve the stability of effect estimates and would increase confidence in attributing causality where there was evidence of an effect.

Our difficulty in attributing causality to observed effects, however, also suggests that novel or complementary approaches to quantitative research are required. Smaller studies can be designed to measure effects at a much finer level of detail and with more specific contextual information. For example, a recent study involving the same CPOE system as was deployed in our intervention trust (but looking at inpatient tests for liver function and plasma gentamicin and vancomycin concentrations) found no change in the volume of test orders but did find other changes (in turnaround time, in information provided with specimens, and in ordering of tests removed from an order set), which suggest that changes might occur at a level of detail that was unobtainable from our data.^17^ Another study of test use in a coronary care unit found reductions in tests ordered when compared with a similar unit and detected no differences in clinical outcomes.^18^ Such smaller studies could be nested within a hospital-wide study.

Much closer linkage with qualitative research would enable a better understanding of quantitative findings. For example, we were not surprised that our study, like almost all previous studies, failed to detect any consistent or plausible beneficial impact of CPOE or PACS on outcomes such as length of inpatient stay. We could interpret the lack of effects on secondary outcomes to mean that, while CPOE systems and PACS might bring important qualitative improvements to the process of clinical care (particularly by making life easier for clinicians once they become familiar with systems),^16^ these benefits are difficult to quantify and detect on a macroscopic (hospital-wide or trust-wide) scale. Our original intention had been to conduct qualitative and quantitative research in parallel, but this was frustrated by delays in obtaining quantitative data. As a consequence, our interviews with users of the systems could not refer to preliminary quantitative findings to elicit explanations for observed effects.

Furthermore, one of our biggest difficulties was in obtaining detailed information on the rollout and use of IT systems during the study period, particularly in the control trusts. Frontline staff in pathology and radiology departments were too heavily burdened with work to respond to requests for information. Higher level staff (managers and consultants) expressed interest in the aims and ultimate success of our study but lacked sufficiently detailed historical knowledge of systems in these departments and referred us to the same frontline staff who had been unable to respond originally. These shortcomings were compounded by “institutional amnesia” resulting from high staff turnover and the demands of more immediate issues.^5^ ^6^

In evaluating the impact of healthcare IT systems, it is conceivable that important qualitative benefits for staff, such as ease of working and reduced workplace stress, are not readily quantifiable. Conversely, quantifiable effects that are not closely linked with qualitative information are difficult to interpret. Healthcare IT has been described as a “diffuse” technology, meaning complex systems comprising multiple components, the implementation of which is as important as their function and the evaluation of which is inherently difficult.^19^ Furthermore, it has been argued that the implementation of such systems must be viewed as a process involving organisational change.^20^ Recent qualitative studies of CPOE^21^ ^22^ ^23^ ^24^ ^25^ ^26^ and PACS^27^ ^28^ ^29^ ^30^ implementations have successfully adopted this “sociotechnical” approach but were not supported by quantitative methods. We have shown the potential of our quantitative methods, but future application must be closely synchronised with qualitative methods.

### Limitations

Although our study benefited from a large number of observations, adjustment for clustering by site and specialty gave rise to large standard errors. Hence, although there seemed to be evidence of potentially important effects for many outcomes, few could be measured with sufficient precision in our final analysis. We restricted our analyses to specialties common to all of the participating trusts, but our results remain susceptible to residual confounding because of differences in case mix between trusts. Confounding was a particular concern in the few instances where the indicator data showed substantial differences between trusts. Inclusion of specialty as a covariate in our regression models to control for differences in case mix, however, did not tend to change our point estimates.

We could not verify data quality, although outpatient data from the commissioning datasets have been assessed as reliable.^31^ Data on pathology tests and radiology examinations were unlikely to contain important omissions as these were obtained directly from pathology and radiology information systems used routinely to manage all requests. Some omissions might have arisen by using local patient identifiers to join these data with data from the commissioning datasets. We had no means to verify the reliability of this process, but the reasonable consistency of our outcome measures between trusts was reassuring.

Our “repeat investigation” measure was a proxy for redundant tests and was dictated by the data available from routine sources. We did not have the level of detail necessary to determine whether tests repeated within this interval were redundant (for example, redundant tests have typically been identified by chart review) or whether they reflected good clinical practice. Our method might not be equally applicable across specialties, but we found no evidence to the contrary by comparing the distributions of times to retest within specialties. Our choice of interval (48 hours) was consistent with that used in other studies.^32^ ^33^ ^34^ Misclassification of tests as redundant when clinically required, and vice versa, would probably have been non-differential and hence would have caused underestimation of underlying effects.

In addition to improved efficiency of delivery of care, modernisation of IT could improve patients’ health outcomes, most obviously through better patient safety. Because our study was designed to take advantage of routinely collected data, we were unable to investigate the impact of CPOE and PACS on health outcomes other than death and overall length of stay.

### Conclusions

Efficiency gains from healthcare IT systems are difficult to quantify. Changes in routinely derived indicators are difficult to interpret and measure. We observed both beneficial and detrimental, or at least unexpected, changes so assumptions of substantial efficiency gains from healthcare IT systems might be unrealistic. Given the large overall benefit that would accrue from small efficiency gains occurring in all trusts across the NHS, further research is justified. Although our underlying methods are promising, quantitative research must be closely allied with qualitative research to provide context and to explain observed changes.

**What is already known on this topic**

The NHS national programme for IT is expected to contribute considerable gains in efficiency
Evidence of quantifiable efficiency gains attributable to healthcare IT systems is limited

**What this study adds**

CPOE and PACS were associated with possible efficiency gains, particularly in ordering of outpatient pathology tests and requests for repeat plain x ray film examination
CPOE and PACS were also associated with possible efficiency reductions
Changes in efficiency from healthcare IT systems based on routinely derived indicators are difficult to quantify because they are difficult to interpret and measure
Assumptions of substantial efficiency gains from healthcare IT systems might be unrealistic

## Notes

Members of the steering group were Aileen Clarke, David King, David Lawrence, Charles Normand, Michael Soljak, and Ken Walton. We thank pathology, radiology, and information technology departments in the trusts and trust managers who provided the data or facilitated this process, and Cristina Masseria, who worked on the project for a year.

Ethical approval: NHS Trent multicentre research ethics committee (MREC/03/4/017) and NHS trust local research ethics committees.

Provenance and peer review: Not commissioned; externally peer reviewed.

## Notes

**Cite this as:** *BMJ* 2008;337:a939

## References

[1] Chaudhry B, Wang J, Wu S, Maglione M, Mojica W, Roth E, et al. Systematic review: impact of health information technology on quality, efficiency, and costs of medical care. Ann Intern Med 2006;144:742-52. 16702590 10.7326/0003-4819-144-10-200605160-00125

[2] Georgiou A, Williamson M, Westbrook JI, Ray S. The impact of computerised physician order entry systems on pathology services: a systematic review. Int J Med Inform 2007;76:514-29. 16567121 10.1016/j.ijmedinf.2006.02.004

[3] Department of Health. Connecting for health. London: DH, 2008 www.connectingforhealth.nhs.uk/

[4] Department of Health. The efficiency map. London: DH, 2005 www.dh.gov.uk/assetRoot/04/11/75/50/04117550.pdf

[5] Hendy J, Reeves BC, Fulop N, Hutchings A, Masseria C. Challenges to implementing the national programme for information technology (NPfIT): a qualitative study. BMJ 2005;331:331-6. 16081447 10.1136/bmj.331.7512.331 PMC1183135

[6] Hendy J, Fulop N, Reeves BC, Hutchings A, Collin S. Implementing the NHS information technology programme: qualitative study of progress in acute trusts. BMJ 2007;334:1360. 17510104 10.1136/bmj.39195.598461.551 PMC1906623

[7] Shadish WR, Cook TD, Campbell DT. Experimental and quasi-experimental designs for generalized causal inference. Boston: Houghton Mifflin, 2002.

[8] Chang BH, Pocock S. Analyzing data with clumping at zero. An example demonstration. J Clin Epidemiol 2000;53:1036-43. 11027937 10.1016/s0895-4356(00)00223-7

[9] Tierney WM, Miller ME, McDonald CJ. The effect on test ordering of informing physicians of the charges for outpatient diagnostic tests. N Engl J Med 1990;322:1499-504. 2186274 10.1056/NEJM199005243222105

[10] Tierney WM, McDonald CJ, Martin DK, Rogers MP. Computerized display of past test results: effect on outpatient testing. Ann Intern Med 1987;107:569-74. 3631792 10.7326/0003-4819-107-4-569

[11] Nitrosi A, Borasi G, Nicoli F, Modigliani G, Botti A, Bertolini M, et al. A filmless radiology department in a full digital regional hospital: quantitative evaluation of the increased quality and efficiency. J Digit Imaging 2007;20:140-8. 17318704 10.1007/s10278-007-9006-y PMC1896266

[12] Weatherburn GC, Bryan S, West M. A comparison of image reject rates when using film, hard copy computed radiography, and soft copy images on picture archiving and communication systems (PACS) workstations. Br J Radiol 1999;72:653-60. 10624322 10.1259/bjr.72.859.10624322

[13] Bryan S, Weatherburn G, Buxton M, Watkins J, Keen J, Muris N. Evaluation of a hospital picture archiving and communication system. J Health Serv Res Policy 1999;4:204-9. 10623035 10.1177/135581969900400405

[14] Watkins JR, Bryan S, Muris NM, Buxton MJ. Examining the influence of picture archiving communication systems and other factors upon the length of stay for patients with total hip and total knee replacements. Int J Technol Assess Health Care 1999;15:497-505. 10874377

[15] Crowe B, Sim L. Implementation of a radiology information system/picture archiving and communication system, and an image transfer system at a large public teaching hospital—assessment of success of adoption by clinicians. J Telemed Telecare 2004;10(suppl 1):25-7. 10.1258/1357633042614339 15603600

[16] Reeves BC, Fulop N, Hendy J, Hutchings A, Collin S, Priedane E, et al. Evaluation of IT modernisation in the NHS. London: National Institute for Health Research, 2008 www.sdo.nihr.ac.uk/sdo442003.html

[17] Westbrook JI, Georgiou A, Dimos A, Germanos T. Computerised pathology test order entry reduces laboratory turnaround times and influences tests ordered by hospital clinicians: a controlled before and after study. J Clin Pathol 2006;59:533-6. 16461564 10.1136/jcp.2005.029983 PMC1860295

[18] Wang TJ, Mort EA, Nordberg P, Chang Y, Cadigan ME, Mylott L, et al. A utilization management intervention to reduce unnecessary testing in the coronary care unit. Arch Intern Med 2002;162:1885-90. 12196088 10.1001/archinte.162.16.1885

[19] Keen J, Bryan S, Muris N, Weatherburn G, Buxton M. Evaluation of diffuse technologies: the case of digital imaging networks. Health Policy 1995;34:153-66. 10153898 10.1016/0168-8510(95)00767-9

[20] Berg M. Implementing information systems in health care organizations: myths and challenges. Int J Med Inform 2001;64:143-56. 11734382 10.1016/s1386-5056(01)00200-3

[21] Aarts J, Doorewaard H, Berg M. Understanding implementation: the case of a computerized physician order entry system in a large Dutch university medical center. J Am Med Inform Assoc 2004;11:207-16. 14764612 10.1197/jamia.M1372 PMC400519

[22] Aarts J, Berg M. Same systems, different outcomes—comparing the implementation of computerized physician order entry in two Dutch hospitals. Methods Inf Med 2006;45:53-61. 16482371

[23] Aarts J, Ash J, Berg M. Extending the understanding of computerized physician order entry: implications for professional collaboration, workflow, and quality of care. Int J Med Inform 2007;76(suppl1):S4-13. 16798068 10.1016/j.ijmedinf.2006.05.009

[24] Callen JL, Westbrook JI, Braithwaite J. The effect of physicians’ long-term use of CPOE on their test management work practices. J Am Med Inform Assoc 2006;13:643-52. 16929035 10.1197/jamia.M2152 PMC1656955

[25] Georgiou A, Westbrook J, Braithwaite J, Iedema R, Ray S, Forsyth R, et al. When requests become orders—a formative investigation into the impact of a computerized physician order entry system on a pathology laboratory service. Int J Med Inform 2007;76:583-91. 16702022 10.1016/j.ijmedinf.2006.04.002

[26] Snyder R, Weston MJ, Fields W, Rizos A, Tedeschi C. Computerized provider order entry system field research: the impact of contextual factors on study implementation. Int J Med Inform 2006;75:730-40. 16326134 10.1016/j.ijmedinf.2005.10.002

[27] Fridell K, Edgren L, Lindskold L, Aspelin P, Lundberg N. The impact of PACS on radiologists’ work practice. J Digit Imaging 2007;20:411-21. 17191101 10.1007/s10278-006-1054-1 PMC3043924

[28] Pare G, Lepanto L, Aubry D, Sicotte C. Toward a multidimensional assessment of picture archiving and communication system success. Int J Technol Assess Health Care 2005;21:471-9. 16262970 10.1017/S0266462305050658

[29] Van de Wetering R, Batenburg R, Versendaal J, Lederman R, Firth L. A balanced evaluation perspective: picture archiving and communication system impacts on hospital workflow. J Digit Imaging 2006;19(suppl 1):S10-7. 10.1007/s10278-006-0628-2 PMC3045172 16763932

[30] Cox B, Dawe N. Evaluation of the impact of a PACS system on an intensive care unit. J Manag Med 2002;16:199-205. 12211345 10.1108/02689230210434934

[31] Outpatient data quality report 2003-03 and 2004-05. London: The Information Centre, 2006 www.ic.nhs.uk/webfiles/publications/outpatientdq/OutpatientDataQualityReport151206_PDF.pdf

[32] Bates DW, Kuperman GJ, Rittenberg E, Teich JM, Fiskio J, Ma’luf N, et al. A randomized trial of a computer-based intervention to reduce utilization of redundant laboratory tests. Am J Med 1999;106:144-50. 10230742 10.1016/s0002-9343(98)00410-0

[33] Bates DW, Boyle DL, Rittenberg E, Kuperman GJ, Ma’luf N, Menkin V, et al. What proportion of common diagnostic tests appear redundant? Am J Med 1998;104:361-8. 9576410 10.1016/s0002-9343(98)00063-1

[34] Weydert JA, Nobbs ND, Feld R, Kemp JD. A simple, focused, computerized query to detect overutilization of laboratory tests. Arch Pathol Lab Med 2005;129:1141-3. 16119987 10.5858/2005-129-1141-ASFCQT

